# Analysis of intra-fractional surface motion during adaptive radiation therapy and relation of internal vs. external position for prostate cancer

**DOI:** 10.1186/s13014-025-02638-3

**Published:** 2025-04-17

**Authors:** Fernanda Macedo-Jiménez, Iris Kalisch, Anna Simeonova-Chergou, Judit Boda-Heggemann, Jens Fleckenstein, Constantin Dreher, Frank A. Giordano, Florian Stieler

**Affiliations:** 1 Department of Radiation Oncology, University Medical Center Mannheim, University of Heidelberg, Heidelberg, Germany; 2 Junior Research Group “Image and Surface guided Radiotherapy”, Mannheim Institute for Intelligent Systems in Medicine (MIiSM), University of Heidelberg, Heidelberg, Germany; 3 DKFZ Hector Cancer Institute at the University Medical Center, Mannheim, Germany; 4 Junior Research Group “Intelligent Imaging for adaptive Radiotherapy”, Mannheim Institute for Intelligent Systems in Medicine (MIiSM), University of Heidelberg, Heidelberg, Germany; 5 Mannheim Institute for Intelligent Systems in Medicine (MIiSM), University of Heidelberg, Heidelberg, Germany

**Keywords:** Surface-guided radiation therapy, Adaptive radiation therapy, Intra-fractional motion, Prostate cancer

## Abstract

**Background:**

Adaptive radiation therapy (ART) allows real-time treatment plan adjustment based on daily anatomical changes but involves a time-consuming workflow. Surface-guided radiation therapy (SGRT) provides precise patient positioning and intra-fractional motion management. This study retrospectively analyses intra-fractional patient motion using SGRT during long-duration radiotherapy (RT) like ART for prostate cancer and further assesses the relation for internal target position measured by cone-beam CT (CBCT) and surface position measured by SGRT.

**Methods:**

Thirty ultra-hypo-fractionated prostate cancer patients (137 fractions) treated with ART on Ethos (version 1.0, Varian Medical Systems, Siemens Healthineers, Palo Alto, CA, USA) using a ring-mounted SGRT system (AlignRT inBore, Vision RT Ltd., UK) were retrospectively analyzed. The mean and standard deviation values of surface positions across three translational axes of up to 60 min of treatment were analyzed. Further, the translational shifts from the second daily CBCT before irradiation and surface position data were compared to evaluate the agreement between internal and surface position. Correlations between CBCT shifts and SGRT data were assessed with the Wilcoxon paired samples test.

**Results:**

The maximum mean (± SD) surface motion was − 2.21 ± 1.27 mm (vertical, at 45 min), 0.22 ± 1.55 mm (longitudinal, at 35 min), and 0.16 ± 0.77 mm (lateral, at 20 min). After the second CBCT shift, the mean (± SD) surface position deviations were − 0.63 ± 1.43 mm (vertical), -0.24 ± 1.63 mm (longitudinal), and 0.05 ± 0.87 mm (lateral) with ranges of 8.30 mm, 10.02 mm, and 6.08 mm on the vertical, longitudinal, and lateral axes, respectively. Significant differences (*p* < 0.05) were found between CBCT and SGRT on the vertical and longitudinal axes.

**Conclusions:**

SGRT revealed a consistent vertical shift over the whole course of long-duration RT and not only for the first minutes of the treatment. Further, SGRT exclusively is not an adequate inter-fractional positioning tool for prostate cancer patients, however additional SGRT-based intra-fractional monitoring can add a value for long duration RT.

**Supplementary Information:**

The online version contains supplementary material available at 10.1186/s13014-025-02638-3.

## Background

Adaptive radiation therapy (ART) enables the precise delivery of radiation therapy (RT) to tumors while minimizing exposure to surrounding organs at risk (OAR) [1]. By continuously assessing and adjusting the treatment plan based on real-time inter- and intra-fractional anatomical changes, ART optimizes treatment accuracy and safety [1].

The ART workflow consists of the following steps: cone-beam computed tomography (CBCT), automatic segmentation and deformable image registration, physicians review, treatment adaptation, quality assurance of the newly generated treatment plan, final approval of ART and dose delivery [2–5]. A recommended step before irradiation involves the acquisition of a “verification” CBCT (vCBCT) followed by co-registration to the initial CBCT to detect intra-fractional anatomical changes due to the necessary time to prepare the ART irradiation. The resulting translational shift is applied to the treatment couch as a last step before beam delivery.

As previously stated in [3–4], the extensive ART workflow translates into comparably longer treatment durations. For ultra-hypo-fractionated prostate cancer, the adaptive workflow at our institution lasted 45–60 min, while conventional RT sessions have been reported to last less than 15 min [6]. This remarkable difference in treatment duration translates into an inevitable change on the internal anatomy of the bladder and rectum [7], making continuous patient monitoring essential during longer treatment durations. However, intra-fractional motion data for prostate cancer treatments currently only covers sessions up to 35 min [4], leaving patient motion during long-duration RT, especially ART sessions, unresolved.

Image-guided RT (IGRT) helps to reproduce the inter-fractional tumor positioning and conditions as planned for treatment. However, daily CBCT image acquisition adds extra radiation exposure to the patient, and most importantly, CBCT cannot provide intra-fractional monitoring during treatment [8–10]. Surface-guided RT (SGRT) uses optical imaging to create a real-time 3D patient surface and avoids the use of extra radiation and external markings [11–15]. SGRT aids in initial positioning, monitors intra-fractional motion, and helps to prevent tumor underdose or added toxicity to the OAR [11, 16]. The American Association of Physicists in Medicine (AAPM) Task Group Report 302 recommends SGRT for treatments directly related to body surfaces, such as breast and head/neck, limb tumors and stereotactic radiosurgery, but does not state the existence of research that supports the use of SGRT for intra-fractional monitoring of deep localized tumors such as the prostate [17].

With missing information on the performance of intra-fractional monitoring during time-intensive treatments for prostate cancer, the questions assessing the patient motion during ART as well as the relation between the internal target position and the surface position remained unanswered and will be assessed in this work.

Part 1 of this study analyses retrospectively the intra-fractional patient motion with SGRT during the course of the time-consuming ART combined with stereotactic body RT (SBRT) prostate ultra-hypo-fractionated irradiation as stated on the Prostate Advances in Comparative Evidence (PACE) trial [18]. In part 2 of this study, we address a known limitation of SGRT for deep-seated tumors like prostate cancer. We analyzed the prostate position due to variable bladder and rectal filling with vCBCT and SGRT. By analyzing the vCBCT data with SGRT surface position, we seek to assess the correlation between internal target position and surface position, providing insight into the suitability of SGRT in monitoring internal changes during prostate RT and ART.

## Materials and methods

### Patient cohort and treatment specifications

This retrospective study analyzed data from 30 patients diagnosed with intermediate- (*n* = 29) and high-risk (*n* = 1) localized prostate cancer treated at the Department of Radiation Oncology, Medical Faculty Mannheim, University of Heidelberg between December 2023 and July 2024, following IRB approval (2024 − 888). Patient cohort characteristics are presented in Table 1, with mean and range values.

**Table 1:** Patient cohort characteristics

Diagnosis	Malignant neoplasm of prostate
Number of patients	30
Age	70.4 (55–83)
Body mass index (BMI)	28.48 (23.05–34.72)
Prostate volume	61.4 cm^3^ (27–112)
Surface-Prostate Isocenter distance	12.10 cm (9.89–16.11)

Treatment planning was performed following the PACE trial, which evaluates the efficacy of ultra-hypo-fractionated SBRT compared to conventional RT and surgery [18]. Patients received 36.25 Gy/40Gy in 5 fractions, with 7.25/8 Gy per fraction delivered on consecutive days. A full bladder and empty rectum institutional protocol was followed for treatment planning (TP) and RT. Planning target volume (PTV) margins were assigned based on the PACE-B and PACE-C protocols. All treatments were delivered using an Ethos linear accelerator (LINAC) in version 1.0 (Varian Medical Systems, Siemens Healthineers, Palo Alto, CA, USA), equipped with a HyperSight CBCT panel (Varian Medical Systems, Siemens Healthineers, Palo Alto, CA, USA) for rapid daily imaging [19–20]. The ART workflow was conducted using Ethos Treatment Management (version 1.0).

### SGRT for ART

**Fig. 1:**
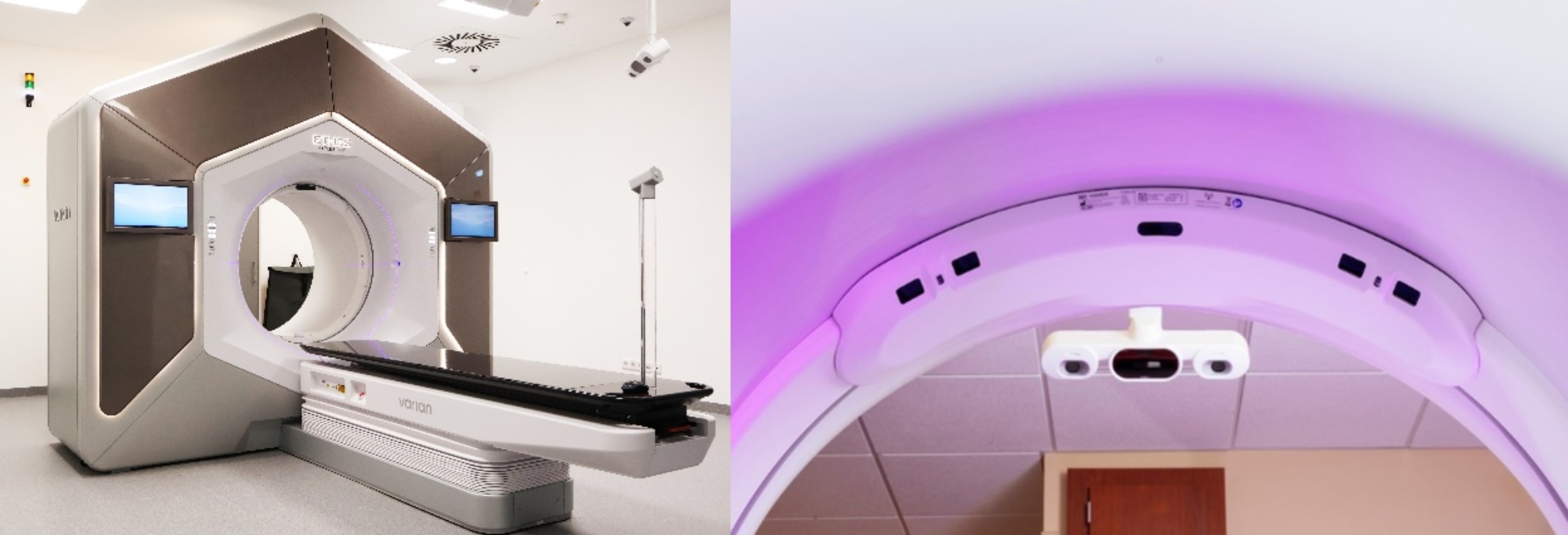
Left panel: Mannheim Ethos LINAC and outer 3D cameras from AlignRT; Right panel: Rear view of AlignRT inBore miniature cameras (VisionRT.com)

The AlignRT® inBore system (Vision RT Ltd., London, UK) is a SGRT technology aimed for patient setup and motion monitoring on ring-based LINACs [21]. The three outer cameras (Fig. 1, left panel) record projected patterns on the patient to continuously acquire 3D surfaces of the patient’s body, while the inBore cameras (Fig. 1, right panel) are miniature cameras with infrared projectors which allow to monitor the patient’s 3-dimensional (3D) surface in treatment position.

The defined region of interest (ROI) was set to cover the anterior and lateral portion of the hips while excluding non-reproducible areas or excessive adipose tissue. Thresholds for real-time deltas (RTDs) were set at ± 3 mm for translational axes and ± 3 degrees for rotational axes.

The SGRT workflow starts by capturing an initial surface reference at the outer isocenter before the adaptive workflow begins on the first treatment fraction. This surface reference is used in subsequent sessions to align the patient at the outer isocenter. The patient is then moved to the treatment isocenter, where the bore-mounted cameras constantly track the patient surface in 6 degrees of freedom (6DoF). While the Ethos LINAC in the current setup only provides translational (vertical, longitudinal and lateral) couch corrections, rotational (pitch, roll and yaw) errors are detected and accounted for during plan adaptation.

The patient is positioned in a head-first, supine position that is tracked by the SGRT system in the following convention for translational motion: vertical (anterior-posterior), longitudinal (cranial-caudal), and lateral (left-right) axes.

Following patient positioning, the ART workflow involves acquiring a daily CBCT. Automatic segmentation models, referred to as “influencer structures” are applied to the daily kV-CBCT within the Ethos Treatment Planning System (TPS). Afterwards, a deformable image registration (DIR) aligns the daily CBCT with the planning CT (pCT), creating a synthetic CT (sCT) [2–4]. The goal of this process is to map the Hounsfield Units (HUs) from the pCT to reflect the anatomy observed in the CBCT for optimal plan generation. Once these structures are reviewed and approved by the physician, two treatment plans are calculated: a scheduled plan based on the reference anatomy and a newly optimized adaptive plan adjusted to the daily anatomical priorities.

Once the ART workflow is completed and the plan selection has been performed by the physician, a pre-irradiation vCBCT is acquired to assess intra-fractional anatomical changes. A rigid image registration between the first CBCT and vCBCT results in a translational “vCBCT shift” that is applied to the treatment couch to correct for the translational changes in the prostate position during the adaptive workflow. During irradiation, interruptions are recommended if RTDs indicate a positional drift out of the ± 3 mm/degrees tolerance set on each translational and rotational directions. Upon session completion, SGRT data including patient motion details are automatically saved as coma-separated values (CSV) and PDF reports.

### Data acquisition and intra-fractional motion/position analysis

Out of 150 treatment fractions given to the patient cohort, 137 fractions were utilized to extract patient motion and position data. Due to technical issues with the LINAC or the AlignRT inBore system and unusual clinical interruptions of the treatment, 13 fractions could not be included in the study. SGRT reports recorded by AlignRT were exported to an Excel spreadsheet (Microsoft Corporation, Redmond, WA, USA) to extract the translational data corresponding to the surface information of the treatment sessions. Ethos Treatment Management provided the translational couch shift calculated from the initial and vCBCT image registration before irradiation.

For part 1 of this study, treatment timelines from SGRT CSV files were matched with Ethos Treatment Management data to track surface position at key treatment stages such as the beginning of ART and at the vCBCT acquisition, identifying the resulting translational shift applied to the treatment couch. MATLAB (R2023a, The MathWorks Inc., USA) was used to process SGRT data, applying a translational vCBCT shift correction to remove the translational shift on the patient surface. Additionally, due to the large amount of surface motion data acquired by the SGRT system, a Savitzky-Golay filter with a window size of 951 points and 1st degree polynomial fitting was applied to reduce breathing motion artifacts. Data processing was finalized by performing a baseline normalization to each data set.

RStudio (version 2024.04.2 + 764, Posit PBC, USA) computed mean and standard deviation values across the three translational axes by taking discrete samples every 5 min during treatment. Mean treatment time was 42.67 ± 7.90 min, with 42% of treatments extending to 50 min or longer, indicating a trend of less sessions with longer time. Mean ($\:\overline x$) and standard deviation (σ) calculations followed standard formulas:

1 $$\:\overline x = \:\frac{1}{n}\sum\nolimits_{i = 1}^n {{x_i}} $$

2 $$\:\sigma \: = \:\sqrt {\frac{1}{{n - 1}}\sum\nolimits_{i = 1}^n {{{({x_i} - \overline x )}^2}} } $$

where $\:n$ is the total number of values, and $\:{x}_{i}$ represents each individual value in the data set.

To assess the 2^nd^ part of this study, which examines the relation between SGRT and CBCT for internal targets, vCBCT shifts and AlignRT positional data were baseline-normalized to identify the time point at which the vCBCT shift was applied to the couch. Subsequently, the positional values in the three translational directions present on the RTDs after the vCBCT shift were taken as the difference from the baseline position. The mean and standard deviation of the differences between SGRT and vCBCT were calculated using RStudio. Since the SGRT and vCBCT vectors presented a non-normally distribution, a statistical agreement between SGRT vectors corresponding to the magnitude and direction of the applied vCBCT shift was tested using a paired-samples Wilcoxon test (*p* = 0.05).

## Results

### Intra-fractional surface motion during ART

As previously mentioned, treatment duration varied across fractions, with only 6.56% (*n* = 9) reaching the maximum of 60 min.

As a result, measurements beyond the 50-minute mark were limited and are directly affected by a low number of measurements. Resulting data up to 45 min of treatment time is considered relevant for discussion. Variations on the BMI of the patient cohort were present, with 40% (*n* = 12) of patients classified as overweight and 36.6% (*n* = 11) of patients classified as obese. The discretely measured intra-fractional surface motion along the three translational planes over the entire treatment duration can be found in the supplemental Table A1.

**Fig. 2:**
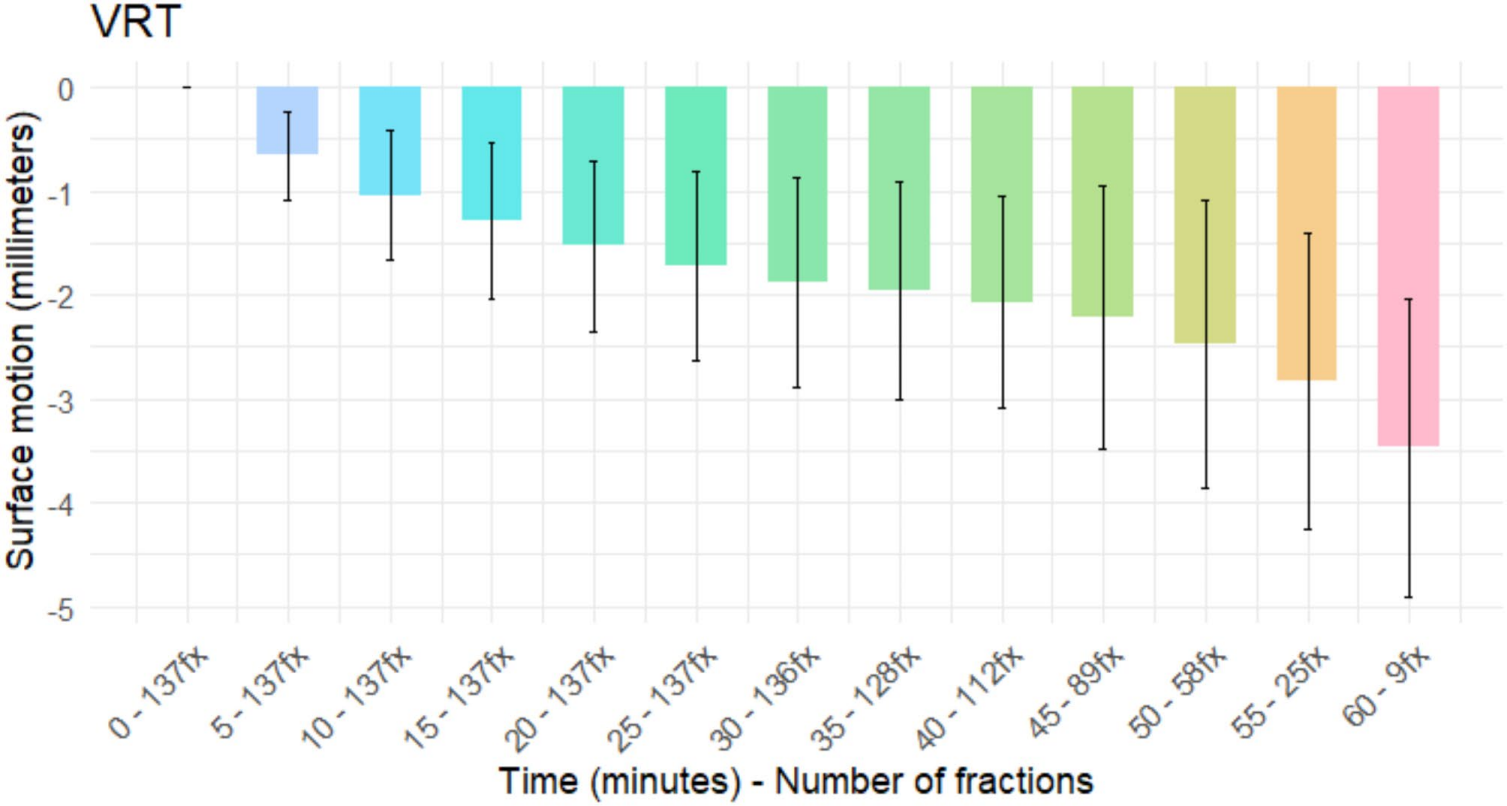
Vertical (VRT) mean and SD during ART combined with SBRT for prostate cancer

Figure 2 presents the results of the mean and standard deviation values of the patient motion on the vertical (VRT) axis with its corresponding number of fractions on each time stamp, revealing an increasing negative deviation over the treatment duration with a maximum value of -2.21 ± 1.27 mm at 45 min (range: -6.93–1.97 mm). The low number of measurements on the remaining treatment time stamps did not show a variation on the decreasing trend.

**Fig. 3:**
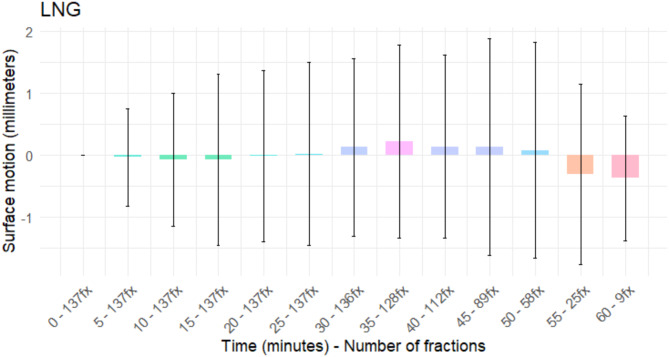
Longitudinal (LNG) mean and SD during ART combined with SBRT for prostate cancer

The obtained results on the patient motion at the longitudinal (LNG) axis are displayed in Fig. 3. An increasing trend during the first half of the treatment time peaks at 35 min with a mean value of 0.22 mm (range: -5.52–6.27 mm) while the standard deviation peaked at 45 min with ± 1.74 mm.

**Fig. 4:**
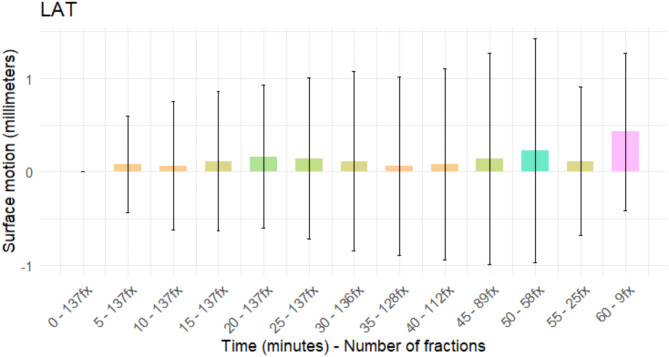
Lateral (LAT) mean and SD during ART combined with SBRT for prostate cancer

Figure 4 presents the mean and standard deviation values of the patient motion on the lateral (LAT) axis. The data does not show any clear trend, and the results up to the 45-minute mark with values of 0.14 ± 1.13 mm (range: -2.96–4.50 mm) on the lateral direction present the lowest variability. Resulting data from 50–60-minute time stamps present a higher deviation affected by a low number of measurements.

### Surface-tumor position correlation and statistical analysis between intra-fractional SGRT and translational CBCT shift

The obtained results regarding the patient surface position in the 3 translational axes after the application of the vCBCT shift are presented in Table 2. In general, the mean position values for all three axes remained below 1 mm magnitude while the standard deviation showed a significant increase, with a maximum value of ± 1.63 mm. The maximum range value was calculated on the LNG axis with 10.02 mm, closely followed by the VRT axis with 8.3 mm.

**Table 2:** Surface-tumor position correlation summary: mean (± SD), maximum, minimum, and range of intra-fractional data obtained from SGRT and translational vCBCT shift

	VRT (mm)	LNG (mm)	LAT (mm)
Mean (± SD)	-0.63 ± 1.43	-0.24 ± 1.63	0.05 ± 0.87
Maximum	3.52	5.88	3.59
Minimum	-4.78	-4.14	-2.49

RTDs vector values correlated to translational vCBCT shift vector in the LAT axis (*p* = 0.7586). No relationship was obtained for VRT (*p* < 0.001) and LNG (*p* < 0.001) axes. Supplemental Table A2 presents the median, inter-quartile ranges (IQR), mean, and p-values obtained from the performed Wilcoxon paired samples test on the vCBCT shifts and SGRT calculated vectors for all the treatment fractions of the patient population.

## Discussion

This work analyzed the intra-fractional motion during ART sessions for prostate cancer to determine the possible patient motion during time-consuming RT and ART. Furthermore, due to missing scientific analyses, we quantified the relation between the surface positioning and the internal target positioning using the example of prostate cancer. Here, we evaluated the translational intra-fractional surface position of the SGRT system and compared it to the gold-standard CBCT image information taken before irradiation after the adaptive workflow.

### Intra-fractional patient motion during ART

As specified in the Materials and Methods section, this study was performed using the early Ethos Treatment Management version 1.0 on the Ethos LINAC, which resulted in overall adaptive workflow times of 33.7 min. However, with a system upgrade after this study to version 2.0 in July 2024, significant improvements in calculation and workflow times have translated into a time reduction of the adaptive workflow of 26%. An overall treatment time reduction with a shorter adaptive workflow would directly reflect a reduced surface motion, especially on the VRT axis. Based on the obtained results, a 26% treatment time reduction reflects a reduced VRT surface motion of -1.95 ± 1.05 mm in our study.

One of our main findings on intra-fractional surface motion involves a significant vertical surface shift over the entire treatment duration and not only for the first few minutes of treatment. This can be related to physical stress and patient relaxation over the full treatment time. According to Cancer Research UK and the American Psychological Association, cancer patients may experience cancer-related stress that reflects as muscular tension during the treatment sessions [22–23]. Similarly, a study by Mannerberg et al. on magnetic resonance-guided RT (MRgRT) found predominant motion of the prostates´center of mass (CoM) on the VRT axis, suggesting that negative displacement on this direction was caused mainly by patient relaxation and bladder volume increase [24]. Since the SGRT system provides surface motion alone, we can assume muscular contraction may occur at the beginning of ART, followed by relaxation as the patient becomes more accustomed to the situation. In addition to this, the variability in the surface motion indicated by the standard deviation suggests that the relaxation time and level is experienced in a different manner among the patient cohort.

The surface motion on the LNG axis remained considerably low (within ± 0.5 mm) throughout most treatment sessions, peaking at 35-minutes but decreasing post-40 min. However, high variability indicated inconsistent and unpredictable motion along this axis. As expected, minimal movements were observed on the LAT axis (below 0.2 mm), highlighting physiological stability achieved at supine position during the treatment sessions.

A study by Apicella et al. examined intra-fractional motion on the pelvic region by utilizing 3D SGRT and found significant movements on the VRT axis with values of -1.55 ± 0.06 mm at 15 min, with minor variations (± 1.00 mm) observed on the LNG and LAT axes [6]. Similarly, Stanley et al. investigated SGRT across various regions such as male and female pelvis, head and neck, breast, and thorax, finding surface displacements of 3.0 ± 3.2 mm, 2.6 ± 3.5 mm, and 2.6 ± 3.4 mm for VRT, LNG, and LAT taken before irradiation under free breathing conditions [25]. While the results from the previous publications present a similar intra-fractional behavior (particularly on the VRT axis) to our findings, the variety of analyzed ROIs as well as the difference in treatment duration resulted in increased surface motion and variability in our study compared to shorter acquisition times (15–35 min).

Taking MRgRT studies into account, Schaule et al. demonstrated stable prostate motion that remained under ± 2 mm in all directions during the first 45 min of treatment [26], while de Muinck Keizer et al. reported information on translational prostate motion with mean values of -1.02 mm, 1.02 mm, and 0.06 mm and an overall high standard deviation on the VRT, LNG, and LAT axes, respectively after 30 min of treatment [27–28]. Our findings on the surface motion of the patient cohort remained within ± 3 mm in all translational directions during the first 45 min of treatment and showed a predominant surface motion on the VRT and LNG directions with high standard deviation values, following a similar trend as MRgRT. Differences in SGRT surface-based tracking and MRgRT internal tracking as well as the implementation of different bladder/rectum regimes for treatment suggest that SGRT and MRgRT provide complementary insights into intra-fractional motion management.

MRgRT studies have also provided significant insights into the dosimetric effects on the target and OARs during extended prostate treatment durations. Unlike our study, this is supported by the acquisition of multiple dose-free magnetic resonance (MR) images during plan adaptation and irradiation that provide sufficient information on the internal organ conditions. Brennan et al. demonstrated the dosimetric effects of using MRgRT for SBRT focal dose intensification by calculating 3 plans from multiple MR scans acquired during the treatment sessions. Their results indicated that the D_95_CTV exceeded 95% in 100% of the patient cohort. They also reported that bladder filling was the only variable that significantly influenced the coverage of the GTV (*p* = 0.03), and stated that the bladder D_0.035 cc_ constraint was exceeded in 1%, 31%, and 45% of times in plans 1, 2, and 3, respectively [29]. Similarly, Mannerberg et al. evaluated the dosimetric effects on the prostate and OARs occurring during adaptive MRgRT with three different PTV-margins. Their results showed a bladder volume increase of 40.9% and a rectum volume difference between −10.9% and 38.8%. The mean CTV D_min_ and PTV D_95%_ dose decreased by 1.1%/2,8% (7 mm PTV margin), 2.0%/2,9% (5 mm PTV margin) and 4.2%/3,1% (3 mm PTV margin). The D_15% Rectum_ and D_mean Bladder_ constraints showed a decrease in dose by 3.6%/12.6% (7 mm), 3.6%/11.8% (5 mm) and 2.6%/10.2% (3 mm) [24]. Nejad-Davarani et al. characterized the dosimetric intra-fractional changes in an MR-only prostate treatment workflow by acquiring MR images with different bladder filling levels. The reported results show a reduction of 11.53% on the PTV D_95%_ between the empty and full bladder [30].

### Surface-tumor position and statistical correlation between intra-fractional SGRT and translational CBCT shift

Analysis of SGRT and vCBCT data from 137 fractions was conducted to assess how well surface position reflects internal prostate position during extended ART sessions. After adjusting for internal tumor changes through vCBCT shift, the LAT surface position showed the closest match to the initial surface setup position at the beginning of the ART workflow (0.05 ± 0.87 mm deviation). However, VRT and LNG deviations were considerably larger with values of -0.63 ± 1.43 mm and − 0.24 ± 1.63 mm, respectively. While the mean surface position difference to the initial setup position after applying the vCBCT shift remained below 1 mm in all 3 directions, the large standard deviation together with the calculated range values of 8.30 mm and 10.02 mm in the VRT and LNG axes, indicate that surface position does not account for internal position variability of the prostate. However, surface guidance helps to avoid rare, larger deviations. The Wilcoxon test revealed a statistically significant correlation between SGRT and vCBCT shifts on the lateral axis, as expected, due to minimal side-to-side movement in the supine position. However, no statistically significant correlation was found on the vertical and longitudinal axes largely due to factors that introduce instability and variability on the surface motion, such as respiratory motion and bladder filling that increases muscular tension on the abdominal area. According to Vision RT, the presence of excessive adipose tissue can cause inaccuracies and motion variability detected by the SGRT system [21]. In our study, 9 patients with a high BMI (overweight-obese) presented inconsistencies between the SGRT and vCBCT shifts higher than ± 0.1 mm in any direction.

A study by Mannerberg et al. compared the use of conventional 3-point laser and SGRT for patient positioning and verified the patient position with the gold-standard technique CBCT on a cohort of 40 prostate cancer patients treated with a hypo-fractionated scheme [31]. Overall, they found small deviations when applying SGRT for patient positioning, with values of 2.2 mm (range 0-9.3), 1.8 mm (range 0-9.6), and 1.1 mm (range 0-5.6) for the VRT, LNG, and LAT axes respectively. The reported values by Mannerberg et al. are similar to the ones encountered in this study on all 3-translational directions. Similarly, Stanley et al. retrospectively compared the CBCT-based corrections of patients positioned with skin tattoos and SGRT for various regions including pelvis/lower extremities, and found an average positioning vector offset of 6 mm with SGRT that agrees with the encountered surface-prostate position range in this study [32]. Walter et al. evaluated patient positioning errors when using SGRT compared to CBCT on 25 patients with different indications including the pelvic region that were treated in either supine or prone position. Their findings on the pelvic region indicate a deviation between CBCT and SGRT of 0.6 ± 1.1 mm on the VRT axis, -1.3 ± 1.4 mm on the LNG axis, and 0 ± 0.1 mm on the LAT axis with no reported range information, showing agreement with our results [33]. Nguyen et al. found that SGRT was effective for tracking lung tumors up to 20 cm below the surface, suggesting SGRT is a reliable alternative to CBCT for thoracic tumors, where surface motion reflects respiratory cycles [34]. However, the findings of their study differ from our experience with prostate tumors, as SGRT did not adequately reflect internal prostate motion, given that prostate shifts are influenced by bladder and rectal changes rather than surface motion. Although the mean prostate position is closer to the surface at 12.10 cm depth compared to the reported lung tumor positions of up to 20 cm underneath the surface, SGRT alone did not offer sufficient accuracy to potentially replace CBCT for internal motion tracking during prostate cancer RT sessions, particularly with extended treatment times related to ART that result in bladder and rectal variations [7]. Another study by Oku et al. recently assessed the accuracy of SGRT for static and moving pelvic region objects using known displacements and found it was accurate within 0.5 mm for static objects and within 1.0 mm for moving objects across all translational directions, aligning with the current study findings [35].

## Conclusion

Our study examined the intra-fractional patient motion for 137 ART treatment sessions with SBRT for 30 prostate cancer patients. The findings highlight a notable constant vertical surface shift over the whole course of treatment and not only for the first minutes of the treatment. Further, the relation between the surface and internal target position after the applied translational vCBCT shifts based on this study revealed that SGRT exclusively is not an adequate inter-fractional positioning tool for prostate cancer patients due to the large range of deviations. However additional SGRT-based intra-fractional monitoring can add a value for long duration radiotherapy.

## Electronic supplementary material

Supplementary table for Tables (PDF 38 kb)

## Data Availability

Data is provided within the manuscript or supplementary information files.

## Abbreviations

3D 3: Dimensional
6DoF: 6 degrees of freedom
AAPM: American Association of Physicists in Medicine
ART: Adaptive radiation therapy
BMI: Body mass index
CBCT: Cone-beam computed tomography
CoM: Center of mass
CSV: Coma-separated value
DIR: Deformable image registration
Hus: Hounsfield Units
IGRT: Image-guided radiation therapy
IQR: Inter-quartile ranges
LAT: Lateral
LINAC: Linear accelerator
LNG: Longitudinal
MR: Magnetic resonance
MRgRT: Magnetic resonance/guided radiation therapy
OAR: Organs at risk
PACE: Prostate Advances in Comparative Evidence
pCT: Planning computed tomography
PTV: Planning target volume
RT: Radiation therapy
RTDs: Real time deltas
sCT: Synthetic computed tomography
SGRT: Surface-guided radiation therapy
TP: Treatment planning
TPS: Treatment planning system
vCBCT: Verification CBCT
VRT: Vertical
