# Supplementary material for: Analysis of intra-fractional surface motion during adaptive radiation therapy and relation of internal vs. external position for prostate cancer
Source: Radiat Oncol. 2025 Apr 17;20:57. doi: 10.1186/s13014-025-02638-3 (PMC12007120; doi:10.1186/s13014-025-02638-3)
Supplement: Supplementary file 1 — Supplementary table for Tables (PDF 38 kb) [file 13014_2025_2638_MOESM1_ESM.docx]

**Supplementary Material**

*Table A1. Intra-fractional surface position of the vertical (VRT), longitudinal (LNG), and lateral (LAT) axes during the treatment duration.*

| Treatment time stamp (min) | Number of fractions | VRT  mean (mm) | VRT  SD (mm) | LNG  mean (mm) | LNG  SD (mm) | LAT  mean (mm) | LAT  SD (mm) |
| --- | --- | --- | --- | --- | --- | --- | --- |
| 0 | 137 | 0.00 | 0.00 | 0.00 | 0.00 | 0.00 | 0.00 |
| 5 | 137 | -0.66 | 0.43 | -0.03 | 0.78 | 0.08 | 0.51 |
| 10 | 137 | -1.04 | 0.62 | -0.08 | 1.07 | 0.07 | 0.69 |
| 15 | 137 | -1.29 | 0.75 | -0.07 | 1.38 | 0.12 | 0.75 |
| 20 | 137 | -1.52 | 0.82 | 0.01 | 1.38 | 0.16 | 0.77 |
| 25 | 137 | -1.73 | 0.91 | 0.02 | 1.48 | 0.15 | 0.86 |
| 30 | 136 | -1.88 | 1.01 | 0.13 | 1.43 | 0.02 | 0.96 |
| 35 | 128 | -1.95 | 1.05 | 0.22 | 1.55 | 0.06 | 0.95 |
| 40 | 112 | -2.07 | 1.02 | 0.13 | 1.47 | 0.08 | 1.02 |
| 45 | 89 | -2.21 | 1.27 | 0.13 | 1.74 | 0.14 | 1.13 |
| 50 | 58 | -2.48 | 1.38 | 0.08 | 1.74 | 0.23 | 1.20 |
| 55 | 25 | -2.83 | 1.43 | -0.31 | 1.45 | 0.12 | 0.79 |
| 60 | 9 | -3.47 | 1.44 | -0.37 | 1.00 | 0.43 | 0.84 |

*Table A2. Median, inter-quartile range (IQR), mean, and p-values values for the patient cohort (n = 30) on the vertical (VRT), longitudinal (LNG), and lateral (LAT) axes.*

|  |  | vCBCT shift | AlignRT inBore | Wilcoxon Test result |
| --- | --- | --- | --- | --- |
| VRT | Median | 1.1 | 1.2 | p-value: 3.626e-4 |
|  | IQR | 1.1 | 1.0 |  |
|  | Mean | 1.3 | 1.32 |  |
| LNG | Median | 0.4 | 0.5 | p-value: 1.845e-6 |
|  | IQR | 1.0 | 0.9 |  |
|  | Mean | 0.53 | 0.56 |  |
| LAT | Median | 0.0 | 0.0 | p-value: 0.7586 |
|  | IQR | 1.4 | 1.3 |  |
|  | Mean | 0.06 | 0.06 |  |
